# Trends in Human Papillomavirus Vaccine Safety Concerns and Adverse Event Reporting in the United States

**DOI:** 10.1001/jamanetworkopen.2021.24502

**Published:** 2021-09-17

**Authors:** Kalyani Sonawane, Yueh-Yun Lin, Haluk Damgacioglu, Yenan Zhu, Maria E. Fernandez, Jane R. Montealegre, Cecilia Ganduglia Cazaban, Ruosha Li, David R. Lairson, Ying Lin, Anna R. Giuliano, Ashish A. Deshmukh

**Affiliations:** 1 Center for Healthcare Data, Department of Management, Policy, and Community Health, UTHealth School of Public Health, Houston, Texas; 2 Center for Health Services Research, Department of Management, Policy, and Community Health, UTHealth School of Public Health, Houston, Texas; 3 Center for Health Promotion and Prevention Research, UTHealth School of Public Health, Houston, Texas; 4 Department of Pediatrics, Baylor College of Medicine, Houston, Texas; 5 Department of Biostatistics and Data Science, School of Public Health, UT Health Science Center at Houston, Houston, Texas; 6 Department of Industrial Engineering, University of Houston, Houston, Texas; 7 Center for Immunization and Infection Research in Cancer, Moffitt Cancer Center, Tampa, Florida

## Abstract

**Importance:**

In the US, safety concern has been identified as a primary barrier to initiating the human papillomavirus (HPV) vaccine. It is unclear if the public’s sentiment concerning HPV vaccine safety aligns with postmarketing vaccine safety surveillance data.

**Objective:**

To perform a parallel assessment of trends in HPV vaccine safety concerns and HPV vaccine adverse event (AE) reporting.

**Design, Setting, and Participants:**

This study was a cross-sectional analysis of the National Immunization Survey (NIS) and Vaccine Adverse Event Reporting System (VAERS). Participants in the NIS were adolescents aged 13 to 17 years. AEs were reported to VAERS by patients, health care clinicians, or other sources. Statistical analysis was performed from October 2020 to May 2021.

**Main Outcomes and Measures:**

Secular trends in HPV vaccine safety concerns and spontaneous AE reporting for HPV vaccination from 2015 to 2018.

**Results:**

Caregivers of 39 364 unvaccinated adolescents with a mean (SD) age of 15.57 (0.08) years (26 996 White adolescents [62.9%], 22 707 male adolescents [56.1%], 11 392 privately insured [62.6%], and 32 674 above the poverty level [79.3%]) reported their reasons for not initiating the HPV vaccine series in the 2015-2018 NIS. Citing safety concerns as the primary reason for not initiating the HPV vaccine series increased from 13.0% (95% CI, 12.1%-14.0%) in 2015 to 23.4% (95% CI, 21.8%-25.0%) in 2018 (*P *for trend < .001), equating to a change from 170 046 to 259 157 US adolescents not initiating the vaccine because of safety concerns. The proportion of parents citing safety concerns as the main reason for HPV vaccine hesitancy increased in 30 states. The largest increases (more than 200%) were observed in California, Hawaii, South Dakota, and Mississippi. During 2015 to 2018, 16 621 AE reports following HPV vaccination were reported to VAERS. The AE reporting rate per 100 000 doses distributed decreased from 44.7 in 2015 to 29.4 in 2018 (*P* for trend < .001). The serious AE reporting rate, including those leading to hospitalizations, disability, life-threatening condition, or death did not change.

**Conclusions and Relevance:**

In this descriptive cross-sectional study, a rise in citing safety concerns was observed among parents with HPV vaccine hesitancy, contrary to the nonserious and serious AE reporting trends. These findings suggest an urgent need to combat the rising sentiment of safety concerns among caregivers to increase HPV vaccine confidence.

## Key Points

**Question:**

Does public sentiment of human papillomavirus (HPV) vaccine safety align with spontaneous HPV vaccine adverse event reporting data?

**Findings:**

This cross-sectional analysis of the 2015 to 2018 National Immunization Survey indicates a 79.9% increase in the proportion of parents who refused the HPV vaccine for their adolescents due to safety concerns. In contrast, estimates from the national vaccine safety surveillance system found that the HPV vaccine adverse event reporting rate per 100 000 doses distributed decreased from 44.7 in 2015 to 29.4 in 2018.

**Meaning:**

These findings suggest an urgent need to combat safety concerns about the HPV vaccine in the US.

## Teaser

This cross-sectional study investigates trends in human papillomavirus (HPV) vaccine adverse event reporting and trends in HPV vaccine safety concerns among caregivers of vaccine-eligible US adolescents from 2015 to 2018.

## Introduction

The human papillomavirus (HPV) vaccine is effective for the prevention of up to 6 cancers (cervical, anal, oropharyngeal, penile, vaginal, and vulvar).^1,2,3,4^ Despite being licensed for over a decade, the HPV vaccine coverage remains suboptimal in the US, with 46% of vaccine-eligible adolescents not up-to-date in 2019.^5^ Furthermore, the coverage varied substantially across states (from nearly 80% in Rhode Island to only 30% in Mississippi).^5^ The coverage among US adults aged 18 to 26 years was also low (only 21.5% in 2018).^6^

The HPV vaccine was demonstrated to be safe and effective in trials before its licensure.^1,4^ Subsequent analyses of the Vaccine Adverse Drug Event Reporting System (VAERS) also established postlicensure safety of the vaccine.^7,8,9^ However, exposure to vaccine misinformation through traditional and social media has created a negative perception of HPV vaccine safety in public.^10,11^ According to a recent national study, safety concern was the top reason for parental lack of willingness for initiating HPV vaccination.^12^ The unfavorable views regarding HPV vaccine safety are contributing to lack in vaccine confidence at an individual level. At a societal level, the collective sentiment of HPV vaccine hesitancy has had an untoward impact on public health policies. In the past, legislative bills proposing the HPV vaccine mandate were overturned, citing vaccine safety concerns.^13^ Despite these repercussions, data documenting HPV vaccine safety perceptions, nationally and across the 50 states, is currently unavailable. Understanding the trends in reasons for failure to HPV vaccinate can provide insights into the extent to which safety-related concerns prevent individuals from receiving the HPV vaccine, and inform the development of interventions to ameliorate this barrier to vaccination.

Data from vaccine adverse event surveillance systems play a critical role in shaping public opinion of vaccine safety. A surge in vaccine safety concerns in the absence of substantive pharmacovigilance data can be indicative of vaccine misinformation in public. Therefore, we performed a parallel assessment of the trends in HPV vaccine safety concerns, as reported in the 2015 to 2018 National Immunization Survey and the trends in nonserious and serious adverse events (AE) reports following HPV vaccination from the 2015 to 2018 VAERS database.

## Methods

The institutional review board of the University of Texas Health Science Centre at Houston deemed this study exempt from review and informed consent because it uses publicly available deidentified data. This cross-sectional study followed the Strengthening the Reporting of Observational Studies in Epidemiology (STROBE) reporting guideline.

### Data Source and Study Criteria

#### Reasons for HPV Vaccine Hesitancy

To examine trends in reasons for HPV vaccine hesitancy, we used the 2015-2018 National Immunization Survey–Teen (NIS–Teen) data. The NIS–Teen is a nationally representative random-dial-digit telephone survey of adolescents aged 13 to 17 years conducted by the Centers for Disease Control and Prevention (CDC). The survey respondents were adult caregivers most knowledgeable of the adolescent’s immunization status. The survey collected information regarding the number of vaccine doses administered. Information on age, sex, race and ethnicity, geographic area of residence, income, and insurance status was self-reported; these data were used in our study to describe the sociodemographic characteristics of the adolescents. Each participant in the NIS was assigned a weight that allowed estimates from the surveyed adolescents to be combined to obtain population estimates (weighted N) that reflected the relative proportions of these groups in the nation as a whole.

For this study, we identified unvaccinated adolescents (received 0 doses of the HPV vaccine) at the time when the survey was administered. Caregivers were then asked if they intend to vaccinate their adolescents in the next 12 months. Parents of unvaccinated adolescents who responded “Not too likely,” “Not likely at all,” and “Don’t know/not sure” were further asked to identify the primary reason for vaccine hesitancy from a list of predefined reasons. If the reason was not listed, the response was solicited in an open-ended manner. In the final data set, all the reasons listed by parents/caregivers were recoded into 28 unique reasons. Additional information regarding the survey methodology and questionnaire is available online.^14^

#### AE Reports

To estimate trends in serious AE reporting, we analyzed the 2015-2018 VAERS database. The VAERS is a national reporting system for monitoring and evaluating vaccine safety in the United States. The system was not designed to examine a causal relationship between the vaccine administered and an AE, but it is useful for signal detection (ie, detecting unusual patterns in AEs). AEs in the VAERS database are voluntarily reported by manufacturers, patients, guardians, health care clinicians, and others. The database contains demographic information of the patient, date of vaccination and date of AE, signs and symptoms of the AE, information regarding the suspected vaccine(s) (including the name of vaccine(s) administered, vaccine type, and manufacturer information), and the outcome of the event. The signs and symptoms reported in VAERS are classified based on a clinically validated standardized methodology, the Medical Dictionary for Regulatory Activities (MedDRA).^15^ A detailed description of VAERS database is available on the VAERS website.^16^

We identified and included all AE reports listing HPV vaccine (including the quadrivalent [4vHPV] and the 9-valent [9vHPV] vaccine) from the 2015 to 2018 VAERS database. The AE was classified as serious (hospitalization or prolonged hospitalization, disability, and death or a life-threatening event) or nonserious events based on the regulatory definition.^17^ To identify if other vaccines were coadministered along with the HPV vaccine, if a patient had an existing medical condition at the time the vaccine was administered, and whether the reported event was based on a social media post (as opposed to a direct report from a patient or a health care professional), we performed manual extraction of this information from free-text field in the reports. All serious AEs were manually evaluated by 4 reviewers (K.S., Y.L., H.D., and A.D.) and cross-validated by a fifth reviewer (Y.Z.) to examine covaccines, existing medical conditions, and social media reports. The analysis was restricted to reports with the US as the country of origin. We used previously published data on the dose distribution of the HPV vaccine in the US to calculate the AE reporting rates.^18^

### Statistical Analysis

We examined the frequency distribution of reasons for HPV vaccine hesitancy during each year from 2015 to 2018 using the NIS–Teen data. Based on the frequency, the top five reasons for HPV vaccine hesitancy were identified (nationally and across 50 states and the District of Columbia). The trends in reasons for not vaccinating were examined using linear regression models. A χ^2^ test was used to compare the proportions for reasons in 2015 vs 2018 across states. All analyses of the NIS–Teen data were adjusted for strata and weights using the SAS SURVEY procedures to account for the complex survey design.

The crude AE reporting rates for the HPV vaccine (per 100 000 doses distributed) were calculated by dividing the number of reports in VAERS by the number of HPV vaccine doses distributed in the US.^18^ Similarly, we examined the reporting rate for serious AE, including those leading to hospitalizations, disability, and death or a life-threatening condition. In the sensitivity analysis, reports of serious AE that were submitted based on online information (personal testimony, blogs, Facebook posts, and tweets) were excluded because these reports were inconsistent in providing details of the patient and the AE.^19^ Trends in the reporting rate were examined using Poisson models accounting for the number of HPV vaccine doses distributed during each year.

Statistical significance was tested at *P* < .05. All analyses were conducted per the analytical guidelines for the NIS–Teen database and VAERS data user guidelines.^14,20^ All analyses were conducted using the SAS statistical software version 9.4 (SAS Institute) from October 2020 to May 2021.

## Results

### Trends in Reasons for Not Receiving the HPV Vaccine

**Figure 1.**
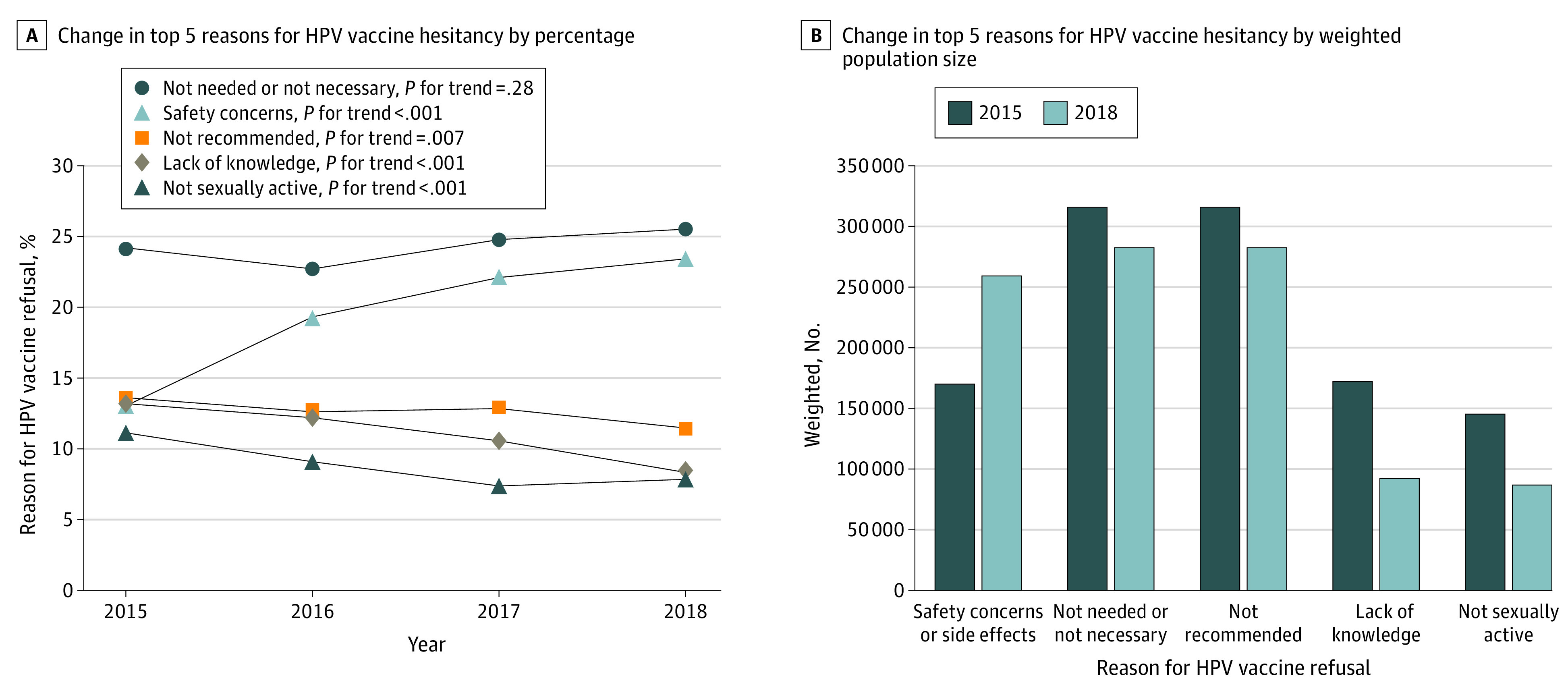
Trends in HPV Vaccine Safety Concerns, National Immunization Survey–Teen, 2015-2018 A, The proportion of US adolescents for whom parents cited safety concerns as the main reason for human papillomavirus (HPV) vaccine hesitancy. The proportions for not needed or not necessary, not recommended, lack of knowledge, and not sexually active are presented. Trends in proportions were examined using linear regression models. B, The population size of adolescents who were unvaccinated due to one of the top 5 reasons. HPV indicates human papillomavirus.

From 2015 to 2018, caregivers of 39 364 unvaccinated adolescents reported their reasons for not initiating the HPV vaccine series (eFigure 1 in the Supplement). In the adolescent cohort with a mean (SD) age of 15.57 (0.08) years, 26 996 (62.9%) were non-Hispanic White, 22 707 (56.1%) were male, 11 392 (62.6%) were privately insured, and 32 674 (79.3%) were from households above the federal poverty line (eTable 1 in the Supplement). The most commonly cited reasons for HPV vaccine hesitancy were “Not needed or not necessary,” “Safety concerns,” “Not recommended,” “Lack of knowledge,” and “Not sexually active,” accounting for 75% of all responses (59 796 of 78 728) (eTable 2 in the Supplement). From 2015 (24.2%) to 2018 (25.5%), there was no change in the proportion of unvaccinated adolescents for whom parents cited “Not needed or not necessary” as their main reason for not vaccinating (Figure 1A). The estimated population size (weighted N) citing “Not needed or not necessary” changed from 315 757 in 2015 to 282 401 in 2018 (Figure 1B). The proportion of unvaccinated adolescents whose parents cited “Safety concerns”’ as the main reason for HPV vaccine hesitancy increased significantly from 13.0% (95% CI, 12.1%-14.0%) to 23.4% (95% CI, 21.8%-25.0%) (*P *for trend < .001), a change in weighted population size from 170 046 in 2015 to 259 157 in 2018. The proportion of adolescents for whom parents cited “Not recommended” (13.6% [95% CI, 12.5%-14.7%] in 2015 to 11.5% [95% CI, 10.3%-12.6%] in 2018; *P *for trend = .007), lack of knowledge (13.2% [95% CI, 12.1%-14.2%] in 2015 to 8.3% [95% CI, 7.4%-9.3%] in 2018; *P *for trend < .001), and not sexually active (11.1% [95% CI, 10.1%-12.2%] in 2015 to 7.9% [95% CI, 6.8%-8.9%] in 2018; *P *for trend < .001) as main reasons for not vaccinating against HPV decreased from 2015 to 2018.

**Figure 2.**
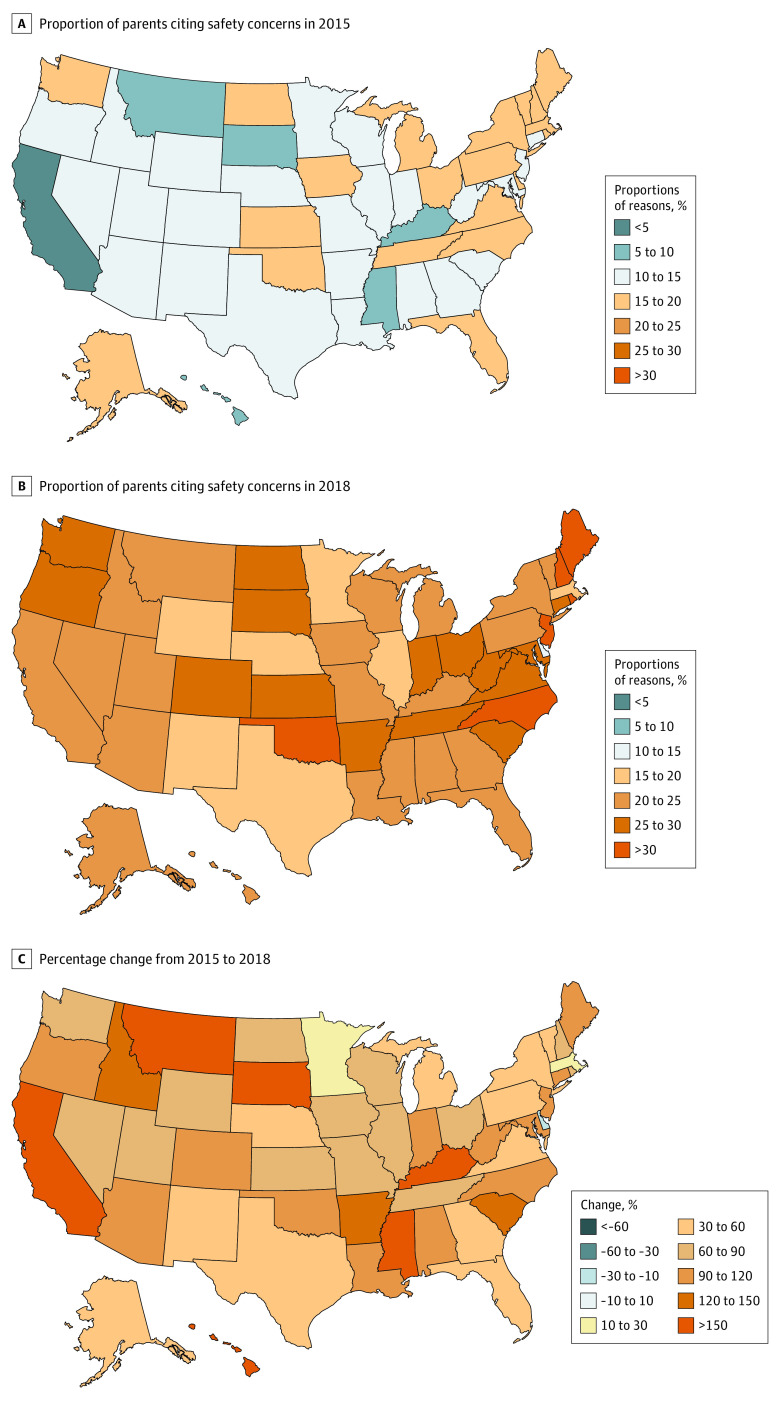
Percentage Change in Human Papillomavirus Vaccine Safety Concerns Across 50 States and the District of Columbia, National Immunization Survey–Teen, 2015-2018 The figure illustrates the percentage change (2015 vs 2018) in the proportion of US adolescents for whom parents cited safety concerns as the main reason for human papillomavirus vaccine hesitancy. Percentage change was examined across 50 states and the District of Columbia.

**Figure 3.**
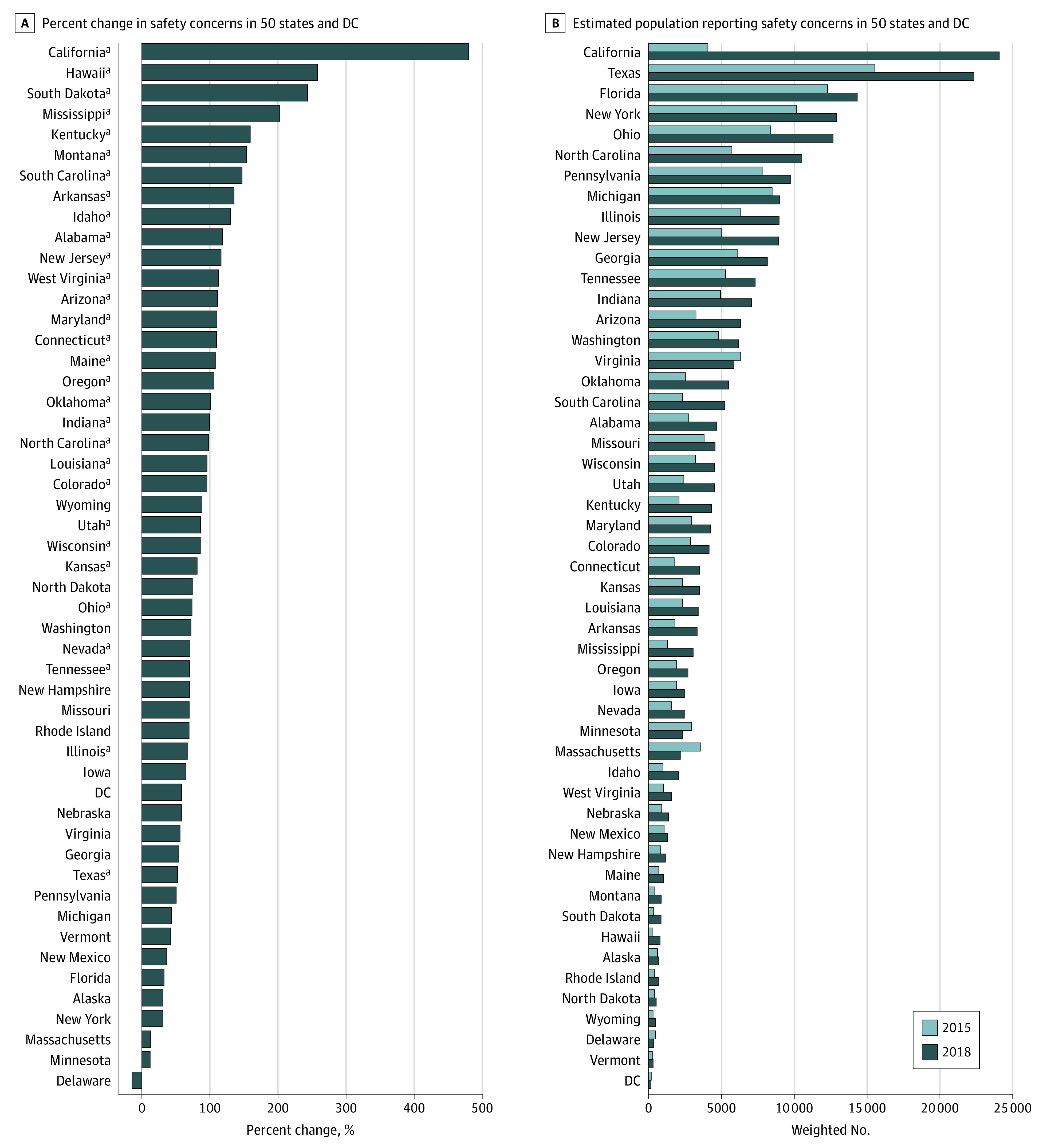
Percentage Change in Safety Concerns and Population Size of Persons Reporting Safety as a Primary Reason for Human Papillomavirus Vaccine Hesitancy in 50 States and the District of Columbia, National Immunization Survey–Teen, 2015 and 2018 A, The percentage change in safety concerns in decreasing order. B, The population size of individuals reporting safety as a primary concern for vaccine hesitancy in 2015 and 2018. ^a^Percentage change was statistically significant.

The US map in Figure 2 illustrates percentage change (2015 vs 2018) in safety concerns as a reason for HPV vaccine hesitancy across the 50 states and the District of Columbia. Overall, a 79.9% increase in the proportion of US adolescents for whom parents cited safety concerns as their primary reason for HPV vaccine hesitancy was observed with significant increases in 30 states. Notably, in California, safety concerns increased from 3.5% (95% CI, 0.2%-6.9%) in 2015 to 20.5% (95% CI, 11.1%-29.8%) in 2018 (*P* < .001) followed by Mississippi (8.1% [95% CI, 1.9%-11.9%] in 2015 to 24.4% [95% CI, 17.0%-31.9%] in 2018; *P* < .001), South Dakota (7.8% [95% CI, 4.0%-11.5%] in 2015 to 26.6% [95% CI, 17.7%-35.6%] in 2018; *P* < .001), and Hawaii (5.8% [95% CI, 2.1%-9.6%] in 2015 to 20.9% [95% CI, 12.3%-29.5%] in 2018; *P* = .002). The percentage change in these 4 states was more than 200%. State-specific percentage change in safety concerns and population size of adolescents who were unvaccinated because of safety concerns are presented in Figure 3A and Figure 3B, respectively. Additional data on the proportions for the top 5 reasons for HPV vaccine hesitancy from 2015 to 2018 are presented in eTable 3 in the Supplement and maps illustrating percentage change (2015 vs 2018) are presented in eFigure 2 in the Supplement. A statistically significant increase in “Not needed or necessary” as the main reason for parental HPV vaccine hesitancy was observed in Delaware (16.3% [95% CI, 9.3%-23.3%] to 34.1% [95% CI, 22.6%-45.7%]), District of Columbia (13.7% [95% CI, 7.0%-20.4%] to 32.7% [95% CI, 17.9%-47.5%]), Florida (18.0% [95% CI, 11.3%-24.7%] to 29.3% [95% CI, 20.8%-37.9%]), Ohio (17.8% [95% CI, 11.7%-24.0%] to 28.4% [95% CI, 20.0%-36.9%]), and Wisconsin (19.8% [95% CI, 13.6%-26.1%] to 34.3% [95% CI, 24.9%-43.7%]).

### Trends in HPV Vaccine AE Reports

During 2015 to 2018, a total of 16 621 AE reports following HPV vaccination were reported to VAERS. Nonserious AEs accounted for 95.4% (15 863) of overall AE reports, while serious AE reports accounted for 4.6% (758) (eTable 4 in the Supplement).

**Figure 4.**
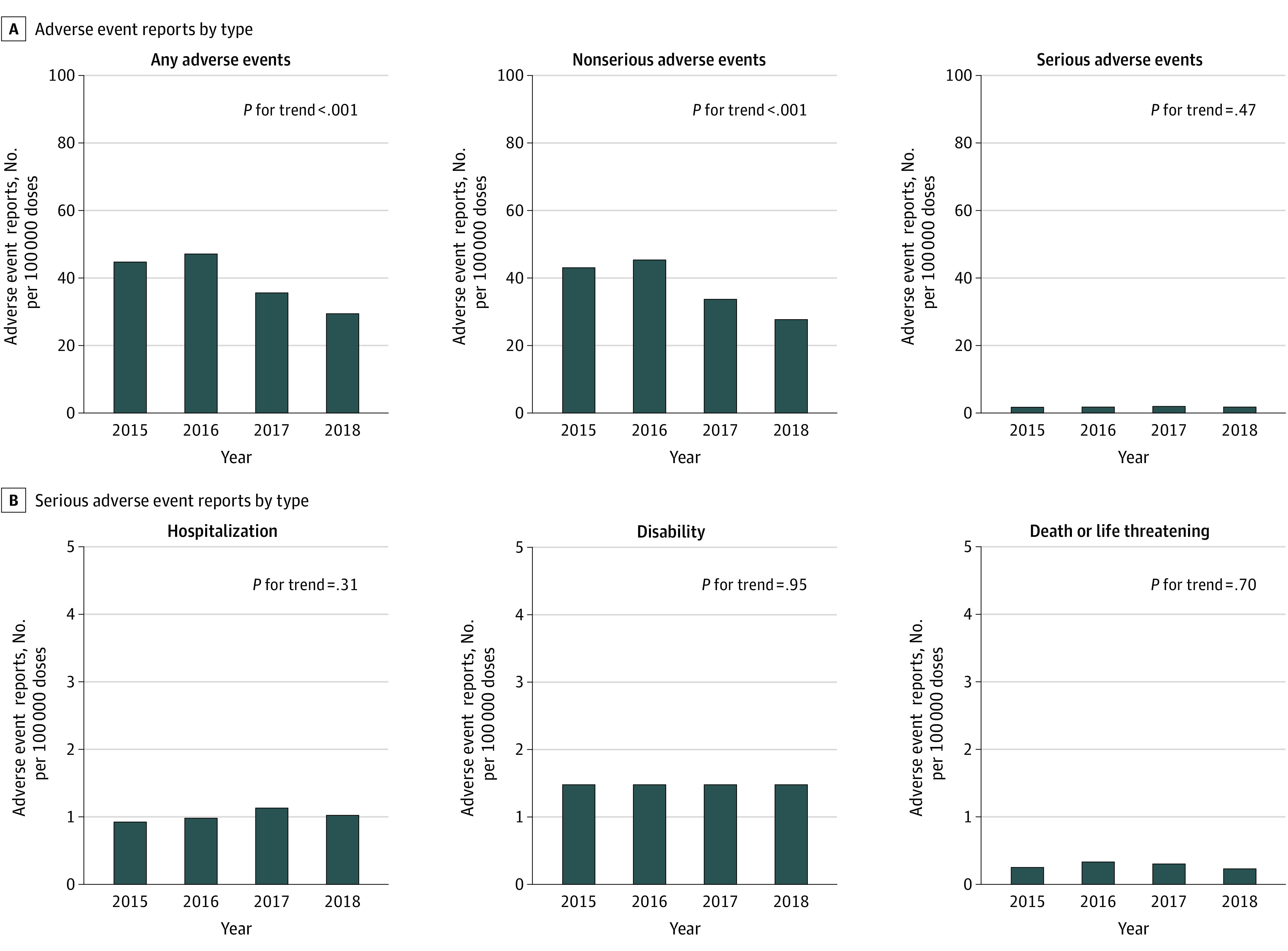
Reporting Rates for Adverse Events Following Human Papillomavirus Vaccination, Vaccine Adverse Event Reporting System, 2015-2018 The figure illustrates reporting rates of adverse events (per 100 000 vaccine doses distributed) following human papillomavirus vaccination. Trends were examined using Poisson models, adjusting for the number of vaccine doses distributed.

The AE reporting rate following HPV vaccination decreased per 100 000 HPV vaccine doses distributed from 2015 to 2018: 44.7 in 2015, 47.1 in 2016, 35.6 in 2017, and 29.4 in 2018 (*P *for trend < .001) (Figure 4A). The reporting rate of nonserious AEs per 100 000 HPV vaccine doses distributed decreased significantly: 43.0 in 2015, 45.3 in 2016, 33.6 in 2017, and 27.6 in 2018 (*P* for trend < .001). The reporting rate of serious AEs per 100 000 HPV vaccine doses distributed did not change during this period: 1.7 in 2015, 1.8 in 2016, 1.9 in 2017, and 1.8 in 2018 (*P *for trend = .47). Figure 4B presents the breakdown of serious AE reported during 2015 and 2018. The reporting rate of serious AE requiring hospitalization per 100 000 HPV vaccine doses distributed did not change: 0.9 in 2015, 1.0 in 2016, 1.1 in 2017, and 1.0 in 2018 (*P *for trend = .31). The reporting rate for disability per 100 000 HPV vaccine doses distributed remained stable from 2015 to 2018 (0.5 across all years; *P *for trend = .95). Similarly, the reporting rate for death or life-threatening conditions per 100 000 HPV vaccine doses distributed remained unchanged from 2015 to 2018: 0.3 in 2015, 0.3 in 2016, 0.3 in 2017, and 0.2 in 2018 (*P *for trend = .70). Trends in reporting rates for hospitalization, disability, and death or life-threatening outcomes were consistent in sensitivity analyses (eFigure 3 in the Supplement).

## Discussion

We examined trends in reasons for HPV vaccine hesitancy and HPV vaccine adverse event reports from 2015 to 2018. During the 4-year period, safety concerns among parents who were HPV vaccine–hesitant increased by approximately 80%. Notably, in 4 states, a greater than 200% increase in citing safety concerns as a primary reason for parental HPV vaccine hesitancy occurred. Nationally, a decreasing trend was observed in the proportion of parents citing other reasons (ie, not recommended, lack of knowledge, and not sexually active). A parallel assessment of VAERS showed a decreasing trend in nonserious AE and no change in serious AE reporting trends during 2015 to 2018.

The trends in reasons for HPV vaccine hesitancy observed in this study are consistent with the literature.^21,22^ For instance, a recent study reported that the number of clinicians recommending the HPV vaccine increased from 27.0% in 2012 to 49.3% in 2018, which might explain the decreasing trend in “Not recommended” as a reason for HPV vaccine hesitancy.^21^ Similarly, a prior study reported a decline in the number of parents who did not initiate the HPV vaccine during 2010 to 2016 owing to concerns of increased sexual activity.^22^ A significant increase in citing safety concerns was also reported during 2010 to 2016 in the study and this upward trend has persisted in recent years based on our analyses.^22^ Furthermore, our data provide additional insights into public perceptions of HPV vaccine safety in the US and to our knowledge, is the first to present trends in all 50 states and DC and concurrently evaluate AE reporting.

The rise in safety concerns noted in our study may have resulted from several reasons. First, it is possible that misinformation related to unsubstantiated AEs of the HPV vaccine on social media and online blogs is increasing mistrust among parents.^11^ At least 2 prior studies have documented a rise in negative content related to the HPV vaccine on social media during 2015 to 2017.^23,24^ Exposure and engagement with antivaccine content is positively correlated with HPV vaccine hesitancy (correlation coefficient = 0.18, *P* = .002).^25^ In a 2017 survey study, parents who had reportedly heard stories about HPV vaccine harms from social media were more likely to refuse the HPV vaccine than the parents who had never heard such stories (odds ratio, 8.9; 95% CI, 4.1-19.3).^11^ Dunn et al^10^ reported a strong correlation between exposure to negative topics (safety concerns and conspiracies) on social media and low HPV vaccination coverage. Collapses of the HPV vaccination programs in Denmark and Japan are prime examples of public mistrust emerging from controversial stories on traditional and social media. The HPV vaccination rates plummeted in Denmark (from approximately 90% pre-2015 to 50% in 2015) and Japan (from approximately 80% pre-2013 to 0.7% in 2013) due to increased misinformation on traditional and social media that misrepresented the risk of vaccine AEs.^26,27,28,29^ The internet has become a major source for parents seeking vaccine information.^30^ Stories about health injuries, disabilities, autism, and even death from receiving the HPV vaccine have been circulating on the social and traditional media that may have misled parents to believe that the vaccine is not safe.^11^ Fear tactics are often used by antivaccine campaigners to dissuade parents from vaccinating. Perceptions that vaccines are unnatural or consist of toxic elements are also often propagated.^31^

The rise in safety concerns was consistent in nearly all states and DC. Particularly, the highest rise in safety concerns was observed in California. A previous study reported that exposure to negative sentiments regarding vaccines was disproportionately higher in California.^32^ Similarly, in Mississippi (where a more than 200% rise in safety concerns was observed), high distrust in parents regarding the HPV vaccine was documented as the most substantial barrier to vaccine uptake.^33^ In another study, approximately one-half of vaccine-eligible individuals’ surveyed from Mississippi reported safety concerns about the HPV vaccine.^34^ Mississippi has the lowest HPV vaccine coverage in the nation; the low vaccine confidence emerging from safety concerns might be contributing to poor HPV vaccine uptake in this state. In the study by Dunn et al, exposure to HPV vaccine safety concerns, misinformation, and conspiracies was a predictor of state-level vaccination coverage (adolescent female coverage correlation=0.82; *P* < .001; adolescent male coverage correlation = 0.70; *P* < .001).^10^ Collectively, these data suggest that vaccine misinformation and antivaccination messages might be contributing to geographic differences in the public’s perception of HPV vaccine safety.

In contrast to the increasing trend in HPV vaccine safety concerns, the reporting rate for any AE following HPV vaccination decreased from 2015 to 2018. A similar decreasing trend was reported during the years 2007 through 2010 in a previous study that evaluated the postlicensure safety of 4vHPV vaccine.^8^ In our study, serious AEs accounted for 4.8% of all reports following the 4vHPV and 9vHPV vaccine, which is similar to the proportion (approximately 3%) of serious AEs reported following the 9vHPV vaccine examined during a similar timeframe (ie, 2014-2017).^35^ Furthermore, reporting rates of serious AE per 100 000 HPV vaccine doses distributed, including reporting rates for events leading to hospitalizations, disabilities, and death or life-threatening outcomes did not change. Trends in serious AE reports following the HPV vaccine are unavailable in the current literature. However, in a previously published study, the proportion of reports with fatal outcomes following the HPV vaccine was 0.7% during 2007 to 2017; the proportion of reports with fatal outcomes in our study was also 0.7%.^36^ Another recent study reported a serious AE reporting rate of 7 per 1 million (or 0.7 per 100 000) 9vHPV doses distributed during 2014 to 2017.^35^ The reporting rate, according to our analysis of VAERS 2015 to 2018 data, is slightly higher (>1.5 per 100 000 doses distributed) because we included both 4vHPV and 9vHPV vaccine reports. From the perspective of public trust, serious AE reports are most critical because they have a very strong influence on personal perception.^37,38^ The summary reports that provide frequency and reporting rates of serious AEs following HPV vaccination are particularly important because they can increase HPV vaccine acceptance.^39^

In the past, countries, including Denmark and Italy, had faced resistance to HPV vaccination due to a rise in vaccine hesitancy. In response to these crises, these countries developed national strategic plans or enforced HPV vaccination policies.^40,41^ Recently, the CDC launched the Vaccine with Confidence Program that has prioritized the issue of vaccine confidence in the US.^42,43^ Additionally, stakeholders, including the American Medical Association have urged social media companies to take action against vaccine misinformation.^44^ Given that state-level HPV vaccine mandates in the past have received backlash in many states, strategies to tackle misinformation should be a public health priority. Efforts at the national level are needed to educate people on the importance of the HPV vaccine. Leveraging social media platforms for communicating the effectiveness and safety of the HPV vaccine toward this effort will be critical. Clinician-targeted interventions, specifically interventions that can help tackle HPV vaccine misinformation and improve vaccine confidence in parents when making decisions pertaining to immunizations are also needed.

### Limitations

This study had a few limitations. Data on trends in safety concerns were examined using the NIS–Teen. Respondents in the NIS–Teen are parents of adolescents (aged 13 to 17 years) eligible to receive the HPV vaccine; therefore, findings may not be generalizable to other vaccine-eligible age groups. Nevertheless, concerns regarding the safety of the HPV vaccine have also been reported in the young adult age group (aged 18 to 26 years) in previous studies.^45,46,47^ Trends in AE reports were examined using the VAERS database. The AEs in the VAERS database are spontaneously reported, and information on critical aspects such as existing disease conditions, co-administered vaccines, and use of medications is often missing or incomplete. Spontaneous AE surveillance systems are also prone to reporting bias; nonserious AEs tend to be underreported whereas reporting sensitivity of serious AEs can vary depending on the outcome.^48^ However, a recent study from Australia found that serious AE reporting rates following HPV vaccination are similar between periods of passive vs active surveillance.^49^ It is important to note that causal inference on the link between the vaccine and reported AEs cannot be drawn from VAERS data for multiple reasons.^50^ Nonetheless, summary data on AE reports generated from VAERS, specifically summaries on serious AEs, can increase HPV vaccine acceptance and trust in the public, which demonstrates the importance of descriptive reports from VAERS.^39^ Finally, our study examined the trends in safety perceptions and AE reporting from 2 separate data sources that currently do not have linkage capabilities; therefore, findings should be interpreted within the context of this limitation.

## Conclusions

In conclusion, concerns regarding safety are rising among HPV vaccine-hesitant parents despite consistent evidence of the vaccine’s safety from prelicensure trials and postmarketing surveillance data. These findings suggest that strategies to combat safety concerns and improve vaccine confidence are urgently needed to expedite the achievement of optimal HPV vaccination coverage in the US.

## Notes

Supplement. **eFigure 1.** Study Sample Flow **eFigure 2.** Percentage Change in Top 5 Reasons For HPV Vaccine Hesitancy Across 50 States And DC, NIS-Teen 2015-2018 **eFigure 3.** Reporting Rates of Serious Adverse Events Following HPV Vaccination Excluding Reports Based On Online Information, VAERS 2015-2018 **eTable 1.** Characteristics of Adolescents, NIS-Teen 2015-2018 **eTable 2.** Reasons for HPV Vaccine Hesitancy, NIS-Teen 2015-2018 **eTable 3.** Top 5 Reasons For HPV Vaccine Hesitancy by States, NIS-Teen 2015-2018 **eTable 4.** Characteristics of HPV Vaccine Adverse Event Reports, VAERS 2015-2018
