# Supplementary material for: Trends in Human Papillomavirus Vaccine Safety Concerns and Adverse Event Reporting in the United States
Source: JAMA Netw Open. 2021 Sep 17;4(9):e2124502. doi: 10.1001/jamanetworkopen.2021.24502 (PMC8449282; doi:10.1001/jamanetworkopen.2021.24502)
Supplement: Supplement. — eFigure 1. Study Sample Flow eFigure 2. Percentage Change in Top 5 Reasons For HPV Vaccine Hesitancy Across 50 States And DC, NIS-Teen 2015-2018 eFigure 3. Reporting Rates of Serious Adverse Events Following HPV Vaccination Excluding Reports Based On Online Information, VAERS 2015-2018 eTable 1. Characteristics of Adolescents, NIS-Teen 2015-2018 eTable 2. Reasons for HPV Vaccine Hesitancy, NIS-Teen 2015-2018 eTable 3. Top 5 Reasons For HPV Vaccine Hesitancy by States, NIS-Teen 2015-2018 eTable 4. Characteristics of HPV Vaccine Adverse Event Reports, VAERS 2015-2018 [file jamanetwopen-e2124502-s001.pdf]

Supplemental Online Content

Sonawane K, Lin YY, Damgacioglu H, et al. Trends in human papillomavirus vaccine safety concerns and adverse event reporting in the United States. *JAMA Netw Open.* 2021;4(9):e2124502. doi:10.1001/jamanetworkopen.2021.24502

eFigure 1. Study Sample Flow

eFigure 2. Percentage Change in Top 5 Reasons For HPV Vaccine Hesitancy Across 50 States And DC, NIS-Teen 2015-2018

eFigure 3. Reporting Rates of Serious Adverse Events Following HPV Vaccination Excluding Reports Based On Online Information, VAERS 2015-2018

eTable 1. Characteristics of Adolescents, NIS-Teen 2015-2018

eTable 2. Reasons for HPV Vaccine Hesitancy, NIS-Teen 2015-2018

eTable 3. Top 5 Reasons For HPV Vaccine Hesitancy by States, NIS-Teen 2015-2018

eTable 4. Characteristics of HPV Vaccine Adverse Event Reports, VAERS 2015-2018

This supplemental material has been provided by the authors to give readers additional information about their work.

© 2021 Sonawane K et al. *JAMA Network Open.*

eFigure 1: Study Sample Flow

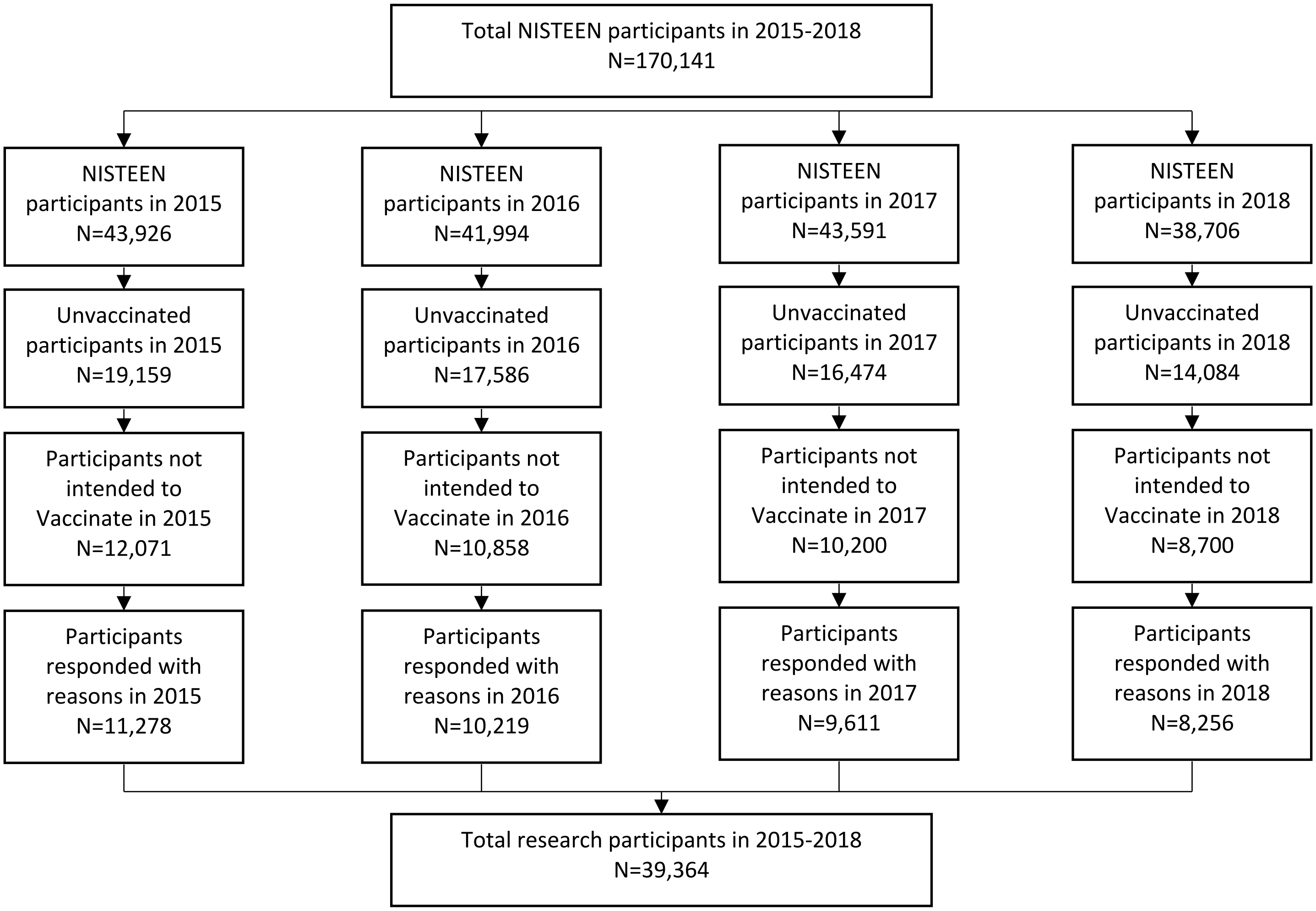

Abbreviation: NIS, National Immunization Survey

© 2021 Sonawane K et al. *JAMA Network Open.*

eTable 1: Characteristics of adolescents, NIS-Teen 2015-2018.

Characteristics	No. of cases	Proportion
**Total**	39,364	100
Age (in years, mean)	15.57	
Male	22,707	56.1
**Race/Ethnicity**		
Non-Hispanic White	26,996	62.9
Non-Hispanic Black	3,186	11.4
Hispanic	5,316	16.7
Other (including multiple race)	3,866	8.8
**Census region**		
Northeast	5,865	14.3
Midwest	8,823	23.5
South	15,759	40.4
West	8,917	21.7
**Insurance**[b]		
Private	11,392	62.6
Medicaid	3,588	26.1
Other	937	5.4
Uninsured	749	5.1
**Poverty status**		
Above poverty (>$75k)	19,049	44.1
Above poverty (≤$75k)	13,625	35.2
Below poverty	4,139	13.7
Unknown	2,551	6.8

© 2021 Sonawane K et al. *JAMA Network Open.*

eTable2: Reasons for HPV vaccine hesitancy, NIS-Teen 2015-2018.

	Survey Year*							
**Reasons**	**2015 N=1,304,608**		**2016 N=1,246,341**		**2017 N=1,121,978**		**2018 N=1,106,441**	
	**%**	**Weighted N**	**%**	**Weighted N**	**%**	**Weighted N**	**%**	**Weighted N**
Not recommended	13.6	177,648	12.6	157,225	12.8	144,116	11.5	126,919
Not needed/Not necessary**	24.2	315,757	22.7	283,060	24.8	278,046	25.5	282,401
Lack of knowledge	13.2	172,082	12.2	152,055	10.6	118,544	8.3	92,182
Not sexually active	11.1	145,242	9.1	113,263	7.4	82,787	7.9	86,813
Not appropriate age	4.4	57,893	4.5	56,641	3.3	37,346	3.7	41,192
Other reason	0.4	5532	1.3	15,782	1.4	15,371	0.9	9410
Costs	0.8	9947	0.9	10,744	0.8	8669	0.8	9282
Safety concern/Side effects	13.0	170,046	19.3	240,801	22.1	247,988	23.4	259,157
Effectiveness concern	0.4	4683	0.3	3737	0.4	3959	0.5	5193
Child fearful	1.5	19,216	1.1	13,698	1.2	13,749	1.3	13,788
Child should make decision	1.3	16,642	1.3	15,722	1.2	13,893	1.1	11,600
College shot^	0.1	636	0.0	474	0.1	964	0.0	42
Don't believe in immunizations	1.2	15,404	1.2	14,820	1.3	14,867	1.7	18,354
Family/Parental decision	3.4	44,375	2.8	34,762	3.0	33,128	4.3	47,467
Handicapped/Special needs/Illness	1.0	13,209	0.7	8952	0.9	10,409	0.9	9855
Religion/Orthodox	0.7	9027	0.6	7588	0.7	8246	0.8	9303
Time	0.2	2696	0.1	1285	0.1	1382	0.1	1306
More info/New vaccine	2.3	29,406	1.9	23,330	2.2	25,080	2.5	27,065
Not available	0.2	2729	0.2	2265	0.1	633	0.1	1342
Not a school requirement	3.7	48,469	3.7	46,129	3.3	36,544	2.4	26,390
Increased sexual activity concern	0.8	10,271	0.8	9345	0.9	10,231	0.9	9926
No OB/GYN	.	NA	0.0	37	.	NA	.	NA
Already sexually active	0.2	2083	0.0	42	0.1	538	.	NA
No doctor or doctor's visit not scheduled	0.6	7924	0.5	5650	0.4	3946	0.4	4844
Child is male	1.8	23,691	1.3	16,332	0.4	4377	0.5	6009
Intended to complete but have not yet/Already planned	.	NA	0.7	9219	0.4	4025	0.4	4216
Difficulty making or getting to appointment/Transportation problem	.	NA	0.3	3384	0.3	3139	0.2	2384

*N's are unvaccinated adolescents for whom parents reported reasons for HPV vaccine hesitancy.
**Not needed or not necessary including up-to-date on vaccine.
^Respondents believe that the vaccination is needed when the adolescent enters university (i.e., at age 18 years)
Abbreviation: NIS, National Immunization Survey; OB/GYN, obstetrician/gynecologist.

© 2021 Sonawane K et al. *JAMA Network Open.*

eFigure 2: Percentage change in top 5 reasons for HPV vaccine hesitancy across 50 states and DC, NIS-Teen 2015-2018.

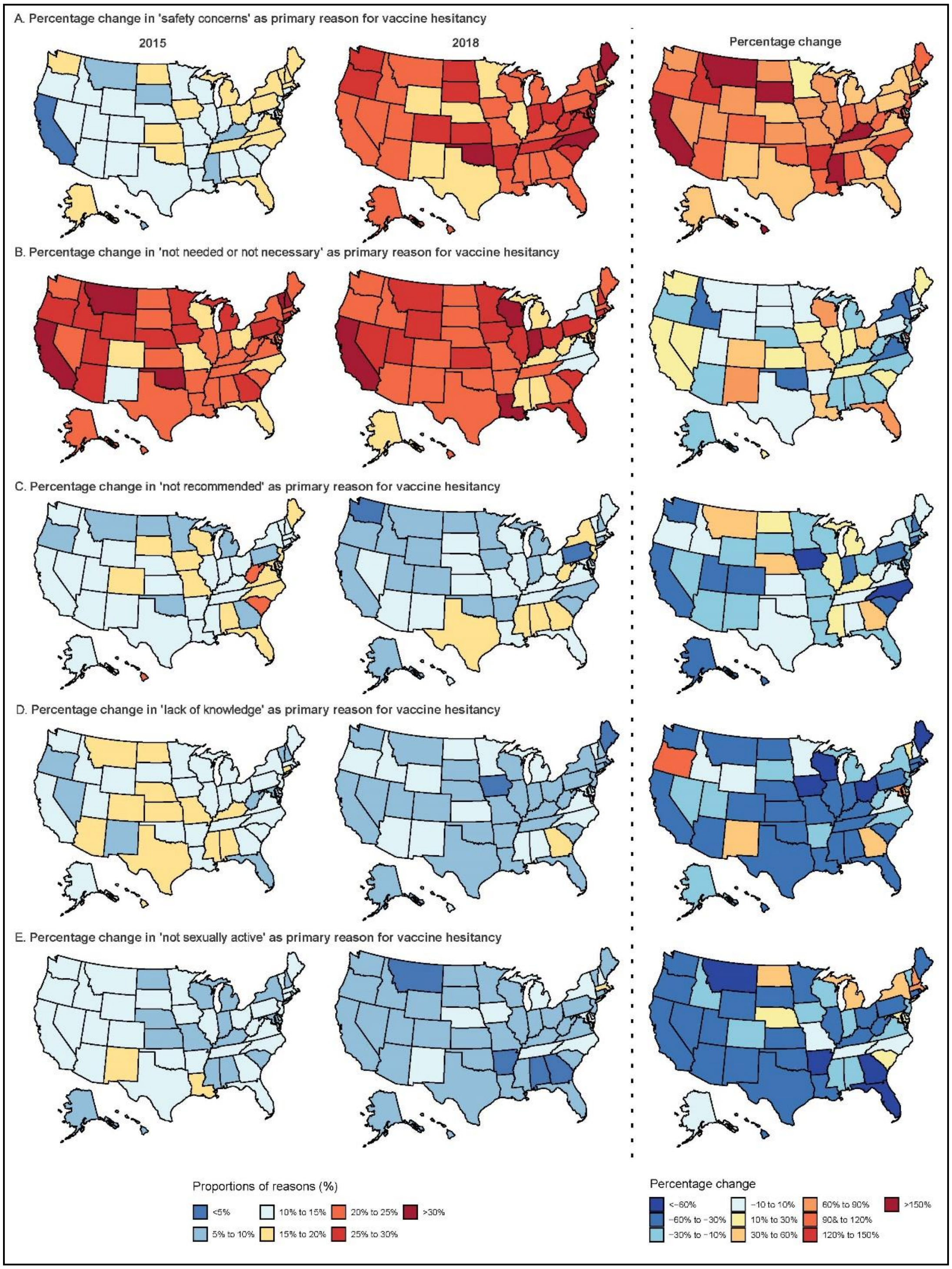

The figure illustrates the percent change (2015 versus 2018) in the proportion of US adolescents for whom parents cited ‘safety concerns’, ‘not needed or not necessary’, ‘not recommended’, ‘lack of knowledge’, and ‘not sexually active’ as the main reason for HPV vaccine hesitancy. Percent change was examined across 50 states and the District of Columbia.

© 2021 Sonawane K et al. *JAMA Network Open.*

eTable 3-1: Top 5 reasons for HPV vaccine hesitancy by states, NIS-Teen 2015-2018.

States	2015				
	Not recommended	Lack of knowledge	Not sexually active	Safety concerns	Not needed or Not necessary including UTD
National	13.6	13.2	11.1	13.0	24.2
Alabama	15.8	17.9	5.8	11.0	22.8
Alaska	13.7	12.3	6.6	16.5	23.7
Arizona	14.8	19.5	10.5	11.4	25.1
Arkansas	10.9	11.1	12.1	11.8	23.5
California	12.1	13.5	10.9	3.5	34.8
Colorado	15.5	15.6	11.3	13.4	18.5
Connecticut	13.5	15.5	12.0	13.8	27.1
Delaware	12.0	14.6	10.8	17.0	16.3
District of Columbia	12.5	13.7	16.5	15.5	13.7
Florida	17.5	9.7	14.1	16.2	18.0
Georgia	9.5	11.5	11.8	13.8	27.2
Hawaii	22.5	17.3	9.8	5.8	21.5
Idaho	10.3	11.6	12.1	10.2	29.7
Illinois	12.6	11.8	10.5	11.3	24.8
Indiana	13.8	12.8	9.8	13.5	25.0
Iowa	15.9	11.6	13.3	15.2	26.4
Kansas	11.4	17.5	9.2	15.5	23.5
Kentucky	11.7	17.8	9.7	9.6	20.5
Louisiana	13.4	14.6	16.0	11.8	23.4
Maine	15.6	12.4	13.0	15.7	21.0
Maryland	19.6	6.8	8.8	12.9	24.1
Massachusetts	12.1	10.2	13.2	16.9	22.9
Michigan	7.0	12.5	7.5	17.4	26.9
Minnesota	10.0	11.1	13.1	13.6	26.4
Mississippi	14.9	16.3	9.5	8.1	23.6
Missouri	15.5	16.4	9.1	12.1	17.1
Montana	5.6	15.6	12.0	8.6	30.2
Nebraska	10.6	17.1	7.8	11.1	29.1
Nevada	13.2	10.0	14.1	14.0	23.4
New Hampshire	12.4	8.4	6.5	18.6	30.2
New Jersey	19.9	10.5	11.9	14.1	26.5
New Mexico	13.8	9.6	18.7	13.1	14.9
New York	14.7	12.9	8.6	16.6	23.3
North Carolina	18.8	13.8	11.3	15.4	18.2
North Dakota	8.7	17.4	6.9	15.1	21.7
Ohio	12.4	14.1	13.4	15.4	17.8
Oklahoma	9.0	14.4	11.5	15.3	30.1
Oregon	9.0	6.6	13.7	12.4	28.6
Pennsylvania	9.4	12.0	13.6	16.2	25.7
Rhode Island	11.7	12.2	9.6	19.3	17.0
South Carolina	23.1	12.0	8.1	11.3	24.1
South Dakota	16.7	12.1	11.4	7.8	21.5
Tennessee	11.0	14.1	11.8	16.3	22.3
Texas	15.0	15.8	11.1	12.1	23.8
Utah	11.3	13.9	11.2	13.1	29.9
Vermont	13.8	8.9	10.3	16.5	30.5
Virginia	15.7	10.8	11.4	17.8	20.9
Washington	10.1	12.3	13.2	16.7	20.4
West Virginia	20.4	8.7	10.7	13.4	22.3
Wisconsin	15.5	14.5	8.2	12.8	19.8
Wyoming	10.1	13.9	12.1	10.1	26.1

Abbreviation: NIS, National Immunization Survey

© 2021 Sonawane K et al. *JAMA Network Open.*

eTable 3-2: Top 5 reasons for HPV vaccine hesitancy by states, NIS-Teen 2015-2018. (Cont)

States	2016				
	Not recommended	Lack of knowledge	Not sexually active	Safety concerns	Not needed or Not necessary including UTD
National	12.6	12.2	9.1	19.3	22.7
Alabama	11.2	11.2	12.3	21.8	20.3
Alaska	7.9	11.8	11.8	19.3	22.2
Arizona	10.7	15.8	8.4	18.1	22.6
Arkansas	16.4	14.2	8.8	16.4	27.0
California	15.0	11.8	9.4	12.7	27.4
Colorado	10.2	6.4	12.7	16.8	31.4
Connecticut	9.5	15.6	7.2	27.2	21.1
Delaware	9.4	9.9	7.1	20.2	24.0
District of Columbia	7.6	14.0	17.3	22.7	16.5
Florida	13.2	13.5	8.1	20.2	16.9
Georgia	10.4	8.5	4.9	25.2	25.7
Hawaii	16.4	9.9	8.5	18.0	19.6
Idaho	14.1	11.6	11.4	16.4	21.2
Illinois	16.3	13.6	8.6	18.8	15.7
Indiana	9.8	12.9	7.8	20.8	25.6
Iowa	8.3	9.6	6.6	19.5	31.1
Kansas	13.3	7.5	10.4	23.6	23.4
Kentucky	16.6	11.6	7.1	19.4	20.4
Louisiana	13.9	9.4	8.6	18.9	21.8
Maine	11.9	8.5	11.5	20.6	25.1
Maryland	11.4	7.8	7.3	19.8	24.9
Massachusetts	9.5	10.1	7.3	29.1	23.7
Michigan	9.0	9.4	7.5	24.4	28.7
Minnesota	10.2	9.9	7.7	26.1	23.0
Mississippi	15.1	11.5	8.5	19.4	24.3
Missouri	10.1	11.0	7.9	16.6	27.7
Montana	5.7	7.8	11.0	12.7	33.2
Nebraska	15.3	11.4	14.3	14.5	24.8
Nevada	11.6	9.2	7.1	20.9	27.0
New Hampshire	14.3	10.0	9.9	18.8	25.1
New Jersey	12.4	13.7	10.8	23.0	16.0
New Mexico	10.9	13.9	7.1	23.6	24.8
New York	16.3	17.5	4.9	17.9	22.0
North Carolina	10.5	14.3	7.8	20.7	26.1
North Dakota	4.7	7.5	6.6	22.7	26.5
Ohio	8.8	14.0	14.7	22.8	15.4
Oklahoma	10.2	14.1	10.1	24.5	15.0
Oregon	10.4	9.2	8.7	15.8	28.8
Pennsylvania	11.4	9.6	9.7	23.0	19.4
Rhode Island	6.3	12.1	6.0	34.5	18.5
South Carolina	14.6	15.7	9.3	18.9	22.4
South Dakota	10.7	11.8	10.1	14.7	28.9
Tennessee	17.0	7.8	14.8	20.4	24.5
Texas	14.9	14.9	8.9	16.3	20.9
Utah	8.2	9.6	11.2	17.2	23.6
Vermont	9.4	11.6	11.4	22.0	22.7
Virginia	18.3	11.7	7.2	18.7	19.6
Washington	8.4	12.2	12.9	20.6	19.5
West Virginia	11.6	12.3	10.8	25.3	24.9
Wisconsin	6.3	11.8	10.8	16.4	24.3
Wyoming	9.6	13.5	15.2	18.3	22.0

Abbreviation: NIS, National Immunization Survey

© 2021 Sonawane K et al. *JAMA Network Open.*

eTable 3-3: Top 5 reasons for HPV vaccine hesitancy by states, NIS-Teen 2015-2018. (Cont)

States	2017				
	Not recommended	Lack of knowledge	Not sexually active	Safety concerns	Not needed or Not necessary including UTD
National	12.8	10.6	7.4	22.1	24.8
Alabama	13.5	13.8	9.9	21.7	27.1
Alaska	5.8	7.3	11.1	24.6	25.8
Arizona	12.8	7.6	7.0	27.2	22.0
Arkansas	14.9	9.9	6.6	25.9	25.2
California	18.7	16.8	7.9	14.4	24.8
Colorado	11.5	9.7	5.9	18.8	21.7
Connecticut	20.3	7.7	5.6	21.3	24.3
Delaware	9.4	11.1	1.4	31.7	25.2
District of Columbia	9.4	8.4	11.7	24.4	14.9
Florida	10.1	15.5	4.3	21.6	30.3
Georgia	11.7	8.0	7.7	16.3	33.2
Hawaii	20.3	9.5	9.2	16.0	26.9
Idaho	8.6	12.9	9.3	18.9	29.5
Illinois	15.3	8.1	3.5	19.6	25.9
Indiana	9.2	5.3	6.5	27.4	27.0
Iowa	8.9	9.1	7.2	24.5	29.7
Kansas	11.2	9.8	7.9	26.4	22.6
Kentucky	9.1	9.7	8.2	28.5	25.2
Louisiana	10.9	16.0	6.1	22.7	19.6
Maine	7.9	6.9	15.7	28.0	22.8
Maryland	13.4	13.1	10.1	20.0	25.3
Massachusetts	18.6	11.8	1.5	30.4	17.2
Michigan	9.0	7.7	10.5	33.5	17.7
Minnesota	8.6	5.5	12.0	21.2	23.2
Mississippi	11.3	12.3	5.1	27.6	22.1
Missouri	15.1	10.5	8.0	20.1	21.7
Montana	9.5	6.7	8.9	26.2	23.2
Nebraska	16.5	4.6	10.8	10.7	36.9
Nevada	9.1	7.8	4.9	26.4	26.4
New Hampshire	11.8	13.8	4.9	21.0	16.4
New Jersey	10.3	10.2	4.6	28.1	20.4
New Mexico	17.0	7.3	3.1	27.4	28.1
New York	16.2	11.4	7.1	19.1	26.4
North Carolina	15.4	12.9	6.6	24.9	20.1
North Dakota	8.2	6.8	3.4	30.3	27.4
Ohio	9.8	8.0	7.6	22.9	27.1
Oklahoma	11.0	9.7	5.2	26.8	25.2
Oregon	17.7	11.9	7.4	23.2	20.1
Pennsylvania	12.3	8.3	7.9	19.1	32.6
Rhode Island	8.8	10.9	6.0	35.4	21.7
South Carolina	16.2	11.0	7.5	27.1	20.2
South Dakota	9.6	6.5	9.1	16.5	32.3
Tennessee	13.7	7.3	11.2	26.0	21.0
Texas	12.2	10.3	8.5	20.7	22.3
Utah	9.2	6.5	9.6	23.7	29.1
Vermont	10.5	4.7	8.4	25.9	26.7
Virginia	16.1	9.6	5.5	16.6	26.7
Washington	8.5	10.5	12.4	23.5	23.5
West Virginia	12.0	9.8	5.4	23.9	26.3
Wisconsin	5.4	13.9	7.9	23.1	20.7
Wyoming	10.0	9.8	9.6	17.0	30.0

Abbreviation: NIS, National Immunization Survey

© 2021 Sonawane K et al. *JAMA Network Open.*

eTable 3-4: Top 5 reasons for HPV vaccine hesitancy by states, NIS-Teen 2015-2018. (Cont)

States	2018				
	Not recommended	Lack of knowledge	Not sexually active	Safety concerns	Not needed or Not necessary including UTD
National	11.5	8.3	7.9	23.4	25.5
Alabama	17.2	11.3	4.7	24.0	17.3
Alaska	9.1	8.8	6.0	21.6	19.2
Arizona	11.0	13.1	6.2	24.2	21.6
Arkansas	8.0	9.1	4.3	27.7	21.4
California	8.0	5.5	6.0	20.5	42.2
Colorado	8.6	8.7	9.5	26.2	24.9
Connecticut	6.9	6.6	8.2	28.9	25.4
Delaware	6.4	10.4	11.6	14.6	34.1
District of Columbia	5.3	7.9	6.5	24.5	32.7
Florida	13.7	5.3	5.2	21.5	29.3
Georgia	15.1	16.0	2.7	21.3	20.7
Hawaii	13.3	11.4	6.3	20.9	24.2
Idaho	9.4	12.0	8.8	23.5	20.4
Illinois	14.3	7.4	6.6	18.9	27.4
Indiana	8.4	6.3	8.3	26.9	30.2
Iowa	5.9	4.0	10.7	25.0	29.6
Kansas	11.0	10.2	5.2	28.0	27.4
Kentucky	13.0	7.4	6.8	24.8	16.3
Louisiana	11.1	7.3	8.7	23.1	30.8
Maine	14.4	1.7	8.3	32.7	24.5
Maryland	15.0	12.1	10.5	27.2	19.9
Massachusetts	10.6	6.3	21.6	19.1	21.8
Michigan	8.0	10.1	10.3	25.0	19.1
Minnesota	8.6	12.0	8.6	15.2	26.3
Mississippi	17.6	11.1	6.8	24.4	18.3
Missouri	12.4	9.6	8.8	20.6	22.7
Montana	8.9	9.4	4.8	21.7	28.4
Nebraska	14.0	9.1	10.1	17.6	24.6
Nevada	11.2	8.3	6.1	24.0	27.0
New Hampshire	5.8	8.3	10.9	31.6	27.6
New Jersey	15.8	9.0	9.5	30.4	19.6
New Mexico	10.1	14.5	11.1	17.8	24.3
New York	15.6	9.3	12.1	21.8	15.0
North Carolina	7.2	10.1	10.7	30.6	14.5
North Dakota	9.6	10.4	9.2	26.2	22.1
Ohio	11.1	5.4	8.3	26.8	28.4
Oklahoma	8.8	9.6	6.3	30.6	20.3
Oregon	8.8	13.9	7.3	25.6	23.4
Pennsylvania	4.7	7.4	9.0	24.4	26.9
Rhode Island	16.9	2.1	2.7	32.6	22.9
South Carolina	10.0	8.4	9.2	28.0	26.8
South Dakota	12.3	10.0	7.7	26.6	21.5
Tennessee	11.9	8.2	12.8	27.7	24.9
Texas	15.6	7.4	7.4	18.4	24.2
Utah	7.5	9.9	5.1	24.3	27.9
Vermont	10.4	10.2	7.4	23.4	19.2
Virginia	14.2	10.3	9.4	27.8	14.0
Washington	4.4	7.0	6.8	28.8	24.4
West Virginia	18.8	6.1	6.2	28.4	19.9
Wisconsin	11.5	5.6	5.8	23.7	34.3
Wyoming	8.2	14.1	7.1	19.1	27.4

Abbreviation: NIS, National Immunization Survey

© 2021 Sonawane K et al. *JAMA Network Open.*

eTable 3-5: Top 5 reasons for HPV vaccine hesitancy by states, NIS-Teen 2015-2018. (Cont)

States	2015 vs 2018 Percentage changes				
	Not recommended	Lack of knowledge	Not sexually active	Safety concerns	Not needed or Not necessary including UTD
National	-15.8*	-36.8*	-29.5*	79.7*	5.5
Alabama	8.5	-36.6	-18.4	118.7*	-24.0
Alaska	-33.7	-28.5	-8.5	31.0	-19.1
Arizona	-25.4	-32.7	-40.6	111.1*	-14.1
Arkansas	-26.8	-17.9	-64.3*	135.5*	-9.0
California	-33.8	-59.4	-45.3	479.9*	21.1
Colorado	-44.3	-44.0	-16.2	95.6*	34.3
Connecticut	-49.2	-57.7	-31.7	109.6*	-6.4
Delaware	-46.6	-28.5	7.6	-14.3†	109.3*
District of Columbia	-57.9	-42.2	-60.9	58.2	139.4*
Florida	-21.5	-45.5	-63.2*	32.7	62.7*
Georgia	59.4	39.8	-76.9*	54.3	-23.8
Hawaii	-40.8	-34.5	-36.1	258.0*	12.6
Idaho	-8.9	3.6	-27.4	130.0*	-31.3
Illinois	13.9	-37.8	-37.7	66.7*	10.3
Indiana	-39.3	-50.9	-14.7	99.5*	20.7
Iowa	-62.7*	-65.1*	-19.9	64.7	12.4
Kansas	-3.8	-41.7	-42.8	81.2*	16.8
Kentucky	10.5	-58.3*	-30.1	159.2*	-20.6
Louisiana	-17.0	-50.4	-45.3	95.8*	31.8
Maine	-7.9	-86.7*	-36.6	108.0*	17.1
Maryland	-23.2	77.6	18.9	110.3*	-17.1
Massachusetts	-12.5	-38.4	63.6	13.0	-5.1
Michigan	15.1	-19.3	37.0	43.8	-29.1
Minnesota	-13.9	7.4	-34.5	12.2	-0.3
Mississippi	17.8	-32.1	-28.4	202.5*	-22.7
Missouri	-20.0	-41.8	-3.1	69.6	32.7
Montana	59.0	-39.9	-60.2*	153.8*	-5.9
Nebraska	32.5	-47.1	29.0	58.1	-15.4
Nevada	-15.3	-16.5	-56.9*	70.7*	15.3
New Hampshire	-53.0	-0.8†	68.0	69.9	-8.7
New Jersey	-20.5	-14.1	-20.3	116.3*	-25.9
New Mexico	-26.7	51.0	-40.8	36.5	62.8
New York	6.6	-27.5	41.7	30.9	-35.6*
North Carolina	-61.6*	-26.5	-5.3	98.2*	-20.0
North Dakota	10.4	-40.2	32.5	74.1	1.9
Ohio	-10.4	-61.9*	-37.7	73.7*	59.5*
Oklahoma	-2.6	-33.0	-44.9	100.6*	-32.5
Oregon	-3.1	110.8	-46.9	106.1*	-18.4
Pennsylvania	-49.5	-38.5	-33.5	50.5	4.7
Rhode Island	44.0	-82.7*	-72.1	69.4	34.9
South Carolina	-56.6*	-30.3	13.1	147.3*	11.6
South Dakota	-26.6	-17.7	-32.9	243.2*	-0.2
Tennessee	8.5	-41.9	8.0	70.2*	12.0
Texas	4.1	-53.2	-33.2	52.4*	1.6
Utah	-34.0	-29.0	-54.9*	86.2*	-6.9
Vermont	-24.2	15.3	-28.3	42.4	-36.9
Virginia	-9.6	-5.0	-17.6	56.2	-33.0
Washington	-56.8	-43.1	-48.9	72.3	19.5
West Virginia	-8.0	-29.3	-42.1	112.3*	-10.5
Wisconsin	-26.1	-61.6*	-29.6	85.9*	73.1*
Wyoming	-18.7	1.4	-41.4	88.4	4.8

Abbreviation: NIS, National Immunization Survey
*Indicates chi-squared test *P*<0.05 for 2015 vs 2018. Chi-squared test was adjusted for complex survey design.
†Estimate may not be reliable due to small sample size.

© 2021 Sonawane K et al. *JAMA Network Open.*

eTable 4-1: Characteristics of HPV vaccine adverse event reports, VAERS 2015-2018.

Report characteristics	2015-2018		
	**Any**	**Serious**	**Non-serious**
**Total**	16621 (100.0%)	758 (4.6%)	15863 (95.4%)
**Age**			
<9 years	191 (1.2%)	2 (0.3%)	189 (1.2%)
9-17 years	6917 (41.6%)	468 (61.2%)	6449 (40.7%)
>17 years	1866 (11.2%)	124 (16.4%)	1742 (11.0%)
Unknown	7647 (46.0%)	164 (21.6%)	7483 (47.2%)
**Sex**			
Female	5863 (35.3%)	548 (72.3%)	5315 (33.5%)
Male	3406 (20.5%)	173 (22.8%)	3233 (20.4%)
Unknown	7352 (44.2%)	37 (4.9%)	7315 (46.1%)
**Product type**			
4vHPV	6661 (40.1%)	447 (59.0%)	6214 (39.2%)
9vHPV	9960 (59.9%)	311 (41.0%)	9649 (60.8%)
**Co-Vaccination reported**	-	42 (5.5%)	-
**Co-Medication reported**	-	157 (20.7%)	-
**Other report sources (Social media, online, etc.)**	-	73 (9.6%)	-
**Report type**			
Death/life-threatening condition	118 (0.7%)	118 (15.6%)	-
Disability	214 (1.3%)	214 (28.2%)	-
Hospitalization	426 (2.6%)	426 (56.2%)	-

Abbreviation: VAERS, Vaccine Adverse Event Reporting System

© 2021 Sonawane K et al. *JAMA Network Open.*

eTable 4-2: Characteristics of HPV vaccine adverse event reports, VAERS 2015-2018. (Cont)

Report characteristics	2015			2016		
	Any	Serious	Non-serious	Any	Serious	Non-serious
**Total**	4645 (100.0%)	177 (3.8%)	4468 (96.2%)	5388 (100.0%)	203 (3.8%)	5185 (96.2%)
**Age**						
<9 years	48 (1.0%)	0 (0 %)	48 (1.1 %)	44 (0.8%)	0 (0.0%)	44 (0.9 %)
9-17 years	1768 (38.1%)	110 (62.2%)	1658 (37.1%)	2015 (37.4 %)	134 (66.0%)	1881 (36.3%)
>17 years	491 (10.6%)	37 (20.9%)	454 (10.2%)	525 (9.7%)	32 (15.8%)	493 (9.5%)
Unknown	2338 (50.3%)	30 (17.0%)	2308 (51.7%)	2804 (52.0%)	37 (18.2%)	2767 (53.4%)
**Sex**						
Female	1420 (30.6%)	126 (71.2%)	1294 (29.0%)	1739 (32.3%)	147 (72.4%)	1592 (30.7%)
Male	841 (18.1%)	39 (22.0%)	802(18.0%)	1022 (19.0 %)	44 (21.7%)	978 (18.9%)
Unknown	2384 (51.3%)	12 (6.8%)	2372 (53.1%)	2627 (48.8%)	12 (5.9%)	2615 (50.4%)
**Product type**						
4vHPV	3645 (78.5%)	159 (89.8%)	3486 (78.0%)	2045 (38.0%)	142 (70.0%)	1903(36.7%)
9vHPV	1000 (21.5%)	18 (10.2%)	982 (22.0%)	3343 (62.0%)	61 (30.0%)	3282 (63.3%)
**Co-Vaccination reported**	-	8 (4.5%)	-	-	9 (4.4%)	-
**Co-Medication reported**		27 (15.3%)			38 (18.7%)	
**Social media report source**	-	23 (13.0%)	-	-	22 (10.8%)	-
**Report type**						
Death/life-threatening condition	-	26 (14.7%)	-	-	38 (18.7%)	-
Disability	-	55 (31.1%)	-	-	53 (26.1%)	-
Hospitalization	-	96 (54.2%)	-	-	112 (55.2%)	-

Abbreviation: VAERS, Vaccine Adverse Event Reporting System

© 2021 Sonawane K et al. *JAMA Network Open.*

eTable 4-3: Characteristics of HPV vaccine adverse event reports, VAERS 2015-2018. (Cont)

Report characteristics	2017			2018		
	Any	Serious	Non-serious	Any	Serious	Non-serious
**Total**	3656 (100.0%)	200 (5.5%)	3456 (94.5%)	2932 (100.0%)	178 (6.1%)	2754 (93.9%)
**Age**						
<9 years	47 (1.3%)	2 (1.0%)	45 (1.3%)	52 (1.8%)	0 (0.0%)	52 (1.9%)
9-17 years	1636 (44.8%)	124 (62.0%)	1512 (43.8%)	1498 (51.1%)	100 (56.2%)	1398 (50.8%)
>17 years	407 (11.1%)	29 (14.5%)	378 (10.9%)	443 (15.1%)	26 (14.6%)	417 (15.1%)
Unknown	1566 (42.8%)	45 (22.5%)	1521 (44.0%)	939 (32.0%)	52 (29.2%)	887 (32.2%)
**Sex**						
Female	1403 (38.4%)	143 (71.5%)	1260 (36.5%)	1301 (44.4%)	132 (74.2%)	1169 (42.5%)
Male	795 (21.8%)	47 (23.5%)	748 (21.6%)	748 (25.5%)	43 (24.2%)	705 (25.6%)
Unknown	1458 (39.9%)	10 (5.0%)	1448 (41.9%)	883 (30.1%)	3 (1.7%)	880 (32.0%)
**Product type**						
4vHPV	699 (19.1%)	91 (45.5%)	608 (17.6%)	272 (9.3%)	55 (30.9%)	217 (7.9%)
9vHPV	2957 (80.9%)	109 (54.5%)	2848 (82.4%)	2660 (90.7%)	123 (69.1%)	2537 (92.1%)
**Co-Vaccination reported**	-	16 (8.0%)	-	-	12 (6.7%)	-
**Co-Medication reported**		54 (27.0%)			38 (21.3%)	
**Social media report source**	-	14 (7.0%)	-	-	14 (7.9%)	-
**Report type**						
Death/life-threatening condition	-	31 (15.5%)	-	-	23 (12.9%)	-
Disability	-	53 (26.5%)	-	-	53 (29.8%)	-
Hospitalization	-	116 (58.0%)	-	-	102 (57.3%)	-

Abbreviation: VAERS, Vaccine Adverse Event Reporting System

© 2021 Sonawane K et al. *JAMA Network Open.*

eFigure 3: Reporting rates of serious adverse events following HPV vaccination excluding reports based on online information, VAERS 2015-2018.

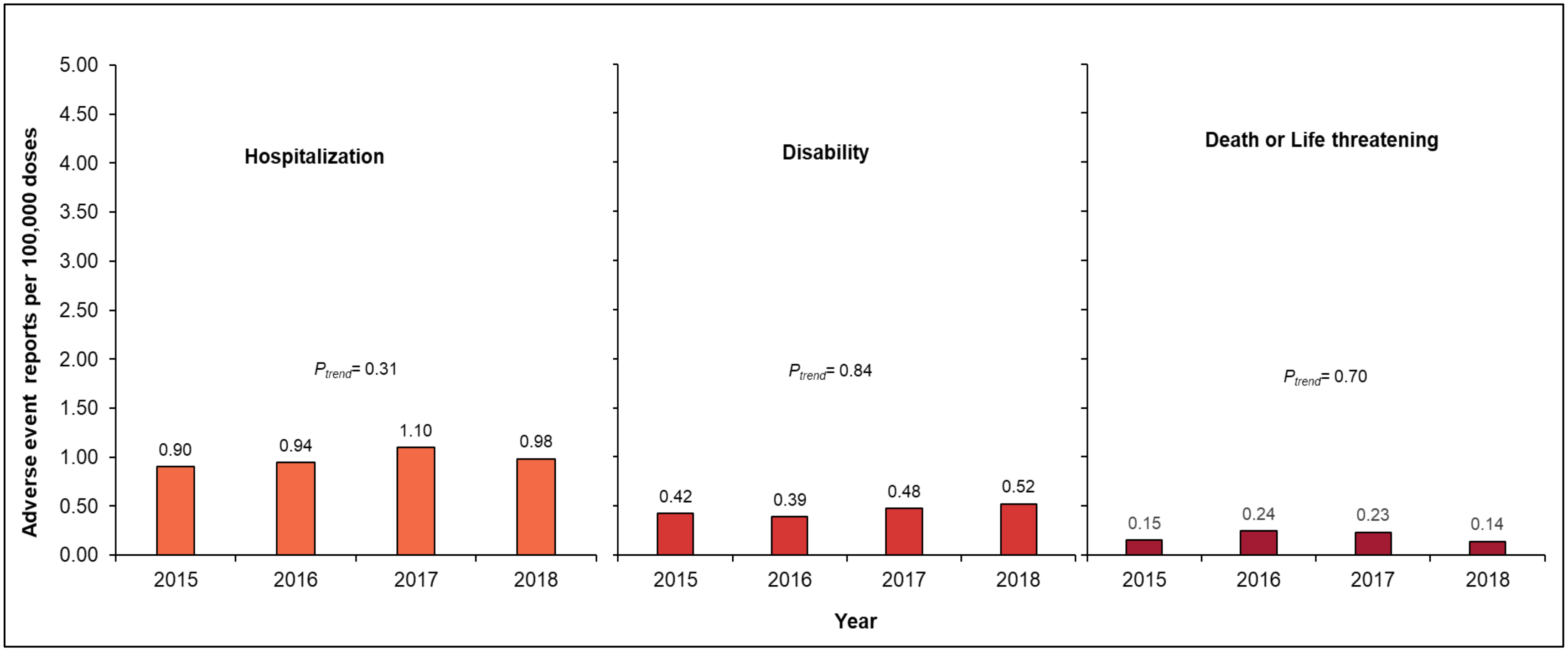

The figure illustrates the findings of sensitivity analyses. Reporting rates for adverse events (per 100,000 vaccine doses distributed) following HPV vaccination were determined after excluding reports that were made to VAERS based on an online source such as a personal testimony, blogs, Facebook posts, or tweets. Trends were examined using Poisson models adjusting for the number of HPV vaccine doses distributed.

Abbreviations: HPV, Human papillomavirus; VAERS, Vaccine adverse event reporting system

© 2021 Sonawane K et al. *JAMA Network Open.*
